# Lucuma Starch-Based Active Packaging Maintains Postharvest Quality of Strawberries During Cold Storage

**DOI:** 10.3390/foods15122093

**Published:** 2026-06-10

**Authors:** Laydy M. Mena-Chacon, Robin Oblitas-Delgado, Angel F. Huaman-Pilco, Pablo Rituay, Krizia Pretell, Eyner Huaman-Huaman, Jonathan Campos

**Affiliations:** 1 Escuela de Posgrado, Programa Doctoral en Ciencias para el Desarrollo Sustentable, Facultad de Ingeniería Zootecnista, Agronegocios, Biotecnología y Ciencias de Datos, Universidad Nacional Toribio Rodríguez de Mendoza de Amazonas, Chachapoyas 01001, Peru; laydy.mena@untrm.edu.pe (L.M.M.-C.); pablo.rituay@untrm.edu.pe (P.R.); krizia.pretell@untrm.edu.pe (K.P.); 2 Grupo de Investigación en Biopesticidas y Bioalternativas para la Protección Vegetal (BIOPEST), Instituto de Investigación para el Desarrollo Sustentable de Ceja de Selva, Universidad Nacional Toribio Rodríguez de Mendoza de Amazonas, Chachapoyas 01001, Peru; robin.oblitas.epg@untrm.edu.pe (R.O.-D.); angel.huaman@untrm.edu.pe (A.F.H.-P.); eyner.huaman@untrm.edu.pe (E.H.-H.); 3 Centro de Investigación Economía Circular y Prospectiva de Agronegocios, Instituto de Investigación en Negocios Agropecuarios, Facultad de Ingeniería Zootecnista, Biotecnología, Agronegocios y Ciencia de Datos, Universidad Nacional Toribio Rodríguez de Mendoza de Amazonas, Chachapoyas, 01001, Peru

**Keywords:** active packaging, lucuma seed starch, *Aloysia citriodora*, strawberry preservation

## Abstract

Sustainable starch-based bioplastics have emerged as promising alternatives to conventional plastics for fresh produce packaging, yet their efficacy in preserving highly perishable fruits remains underexplored. Strawberries cv. San Andreas, prone to rapid postharvest deterioration, require packaging that balances moisture retention and gas exchange to maintain quality. This study developed lucuma seed starch-based bioplastics incorporated with free (EO) or microencapsulated (EOM) lemon verbena essential oil and evaluated their performance during 16 days of refrigerated storage (4 °C) compared to non-active bioplastic (Control) and commercial low-density polyethylene (LDPE). Microencapsulation enhanced the stability and controlled release of bioactive compounds. The EOM treatment reduced weight loss to 12.81% (vs. 18.25% in Control and 6.29% in LDPE), while preserving firmness at 3.87 N (vs. 2.19 N in LDPE). Strawberries packaged in both EO and EOM exhibited complete suppression of visible decay (0% incidence) throughout storage, in stark contrast to LDPE (57.34% incidence). The EOM system also maintained higher levels of total phenolics (205.51 mg GAE/100 g FW), antioxidant capacity (289.05 µmol TE/100 g FW), and anthocyanins compared to LDPE and Control treatments. These findings demonstrate that lucuma seed starch bioplastics containing microencapsulated lemon verbena essential oil represent a sustainable and functional packaging strategy to extend shelf life and preserve the quality of highly perishable strawberries during refrigerated storage.

## 1. Introduction

The increasing demand for sustainable food systems has intensified the search for eco-friendly packaging materials capable of reducing plastic waste while preserving the quality and safety of fresh produce. In this context, starch-based bioplastics derived from agro-industrial residues have emerged as promising alternatives to conventional petroleum-based plastics due to their renewability, biodegradability, and film-forming capacity. In particular, the valorization of fruit processing by-products as starch sources represents a key strategy within the circular economy, enabling waste reduction while generating value-added materials for food applications [1,2]. Indeed, fruit seed starches—such as those from mango, avocado, and lucuma—have demonstrated favorable physicochemical and functional properties for developing biodegradable packaging systems [3,4,5,6,7].

However, beyond sustainability, the effectiveness of packaging systems for fresh produce largely depends on their ability to regulate mass transfer phenomena, particularly the exchange of water vapor and respiratory gases such as oxygen (O_2_) and carbon dioxide (CO_2_). These processes directly influence fruit respiration, transpiration, and microbial growth, which ultimately determine postharvest quality and shelf life [8,9,10]. Therefore, achieving a balanced barrier performance is critical: excessive permeability may accelerate dehydration and deterioration, whereas overly restrictive barriers can induce anaerobic conditions and negatively affect fruit metabolism.

Among emerging starches, lucuma (*Pouteria lucuma*) seed starch has gained attention as a novel and underutilized raw material for biodegradable packaging applications. Lucuma seeds contain high starch content (≈84%) and exhibit desirable properties such as high-water absorption, swelling capacity, and good film-forming ability [11,12]. Furthermore, lucuma seed starch presents a relatively high amylose content (35.9%; [13]), exceeding the values commonly reported for conventional cassava or rice starches (15–25%) [14,15]. High-amylose starches are known to form stronger and more cohesive polymer networks with improved water vapor barrier capacity, tensile strength, and gas barrier performance, all of which are highly desirable attributes for fresh produce packaging [16,17,18].

Additionally, the fine granule morphology of lucuma seed starch (7.41 ± 0.48 µm) may enhance polymer interactions and facilitate the incorporation and retention of bioactive compounds within the polymer matrix [19,20]. Its moderate gelatinization temperature (≈65 °C) also suggests favorable processing characteristics while maintaining thermal stability suitable for film formation [21]. Collectively, these characteristics position lucuma seed starch as a promising candidate for the development of functional and active biodegradable packaging systems. In this regard, lucuma seed starch appears to occupy an intermediate position between conventional starches and high-amylose legume starches, potentially providing a balanced combination of mechanical strength, water resistance, and processability. Such characteristics are particularly advantageous for active packaging systems requiring both structural integrity and controlled release of incorporated bioactive compounds.

To further enhance the functionality of these materials, the incorporation of natural bioactive compounds such as essential oils has been widely explored. Essential oils exhibit strong antimicrobial and antioxidant properties, contributing to the inhibition of postharvest pathogens and the preservation of food quality [22,23]. In particular, *Aloysia citrodora* (syn. *Aloysia triphylla*, *Lippia citrodora*; https://gni.globalnames.org; https://powo.science.kew.org), commonly known as lemon verbena, cedrón, or lippia essential oil has demonstrated significant antifungal activity against *Botrytis cinerea* [24,25,26], one of the main causes of postharvest decay in strawberries, as well as the ability to preserve physicochemical attributes such as weight, acidity, and antioxidant compounds during storage [25].

Nevertheless, the direct incorporation of essential oils into polymer matrices presents several limitations, including high volatility, rapid release, and potential sensory alterations in food products [23]. In this context, microencapsulation has emerged as an effective strategy to improve essential oil stability, protect volatile compounds, and enable controlled release while maintaining antimicrobial effectiveness [27,28]. Recent studies have shown that microencapsulated lemon verbena essential oil can improve flexibility and UV-protection properties of starch-based bioplastics while preserving their mechanical integrity, supporting its relevance for active food packaging applications [26,29].

Despite these advances, a critical gap remains in the functional validation of starch-based active packaging systems under realistic postharvest conditions. Most previous studies have focused primarily on material characterization or in vitro antimicrobial assays, whereas relatively few investigations have evaluated the actual effectiveness of these systems in preserving highly perishable fruits during storage. Furthermore, the integration of microencapsulated essential oils into lucuma starch-based matrices and their influence on mass transfer regulation and fruit quality preservation remain poorly understood.

Strawberries (*Fragaria* × *ananassa*) are highly perishable fruits characterized by high respiration rates, elevated water activity, and high susceptibility to fungal decay, particularly caused by *Botrytis cinerea* [10,30]. Their rapid postharvest deterioration, driven by moisture loss, gas exchange, and microbial proliferation, makes them an ideal model to evaluate the effectiveness of active packaging systems [31,32]. In addition, the ‘San Andreas’ cultivar is widely recognized for its high commercial productivity and representative postharvest behavior among commercial strawberry cultivars [33], supporting its suitability for evaluating novel active packaging systems.

In this context, the present study aimed to evaluate, at laboratory scale, the effectiveness of a lucuma seed starch-based active packaging system incorporating free and microencapsulated lemon verbena essential oil in maintaining the postharvest quality of strawberries during cold storage. This work provides novel insights into the synergistic effect of biopolymer-based matrices and controlled-release systems, contributing to the development of sustainable and functional packaging solutions for fresh produce.

## 2. Materials and Methods

### 2.1. Materials and Reagents

Strawberry fruits (‘San Andreas’) were harvested early in the morning in Luya, Amazonas, Peru (6.1540° S, 77.9445° W; 2313 m a.s.l.; WGS84) and immediately transported to the Plant Physiology Laboratory of the National University Toribio Rodríguez de Mendoza de Amazonas (UNTRM). Upon arrival, fruits were selected based on uniformity of size, ripeness, and absence of visible defects. Fruits corresponded to ripening stage 3, characterized by red coloration extending to the middle region of the fruit with pinkish hues surrounding the calyx. The respiration rate at harvest was 185.4 ± 5.1 mg CO_2_·kg^−1^·h^−1^.

Lucuma seed starch-based bioplastics containing free essential oil (EO) or microencapsulated essential oil (EOM) of lemon verbena were evaluated in this study. The formulations were selected from preliminary optimization trials previously conducted by the authors, considering their film-forming capacity, structural stability, and barrier properties relevant to fresh produce preservation [13].

Microencapsulated EO was obtained under previously optimized spray-drying conditions designed to improve the stability and controlled release of volatile bioactive compounds [26]. All chemicals and reagents used were of analytical grade and purchased from Sigma-Aldrich^®^ (St. Louis, MO, USA).

### 2.2. Preparation of Microencapsulated Essential Oil (EOM)

Microencapsulation of lemon verbena EO was performed using a laboratory-scale spray dryer (LabPlant SD-06AG, Huddersfield, UK) equipped with a 1.0 mm spray nozzle, following the methodology described by Mena-Chacon et al. [26]. Soy lecithin and dextrin were used as emulsifier and wall material, respectively. Briefly, lecithin (1 g) was dispersed in distilled water (30 g) at 50 °C under magnetic stirring (350 rpm for 15 min). After cooling to 30 °C, EO (1 g) was added and homogenized at 1500 rpm for 3 min. Separately, dextrin was dispersed in distilled water at 50 °C and stirred for 3 h to achieve a 1:5 core-to-wall material ratio (Ct). Subsequently, the EO emulsion was incorporated into the dextrin solution and homogenized at 1500 rpm for 3 min before spray drying.

Spray drying conditions were standardized as follows: feed solution concentration of 5% (*w*/*v*), feed temperature of 42 ± 2.5 °C, inlet air temperature of 194 ± 1 °C, feed rate of 5 mL/min, atomization pressure of 1.25 bar, and ventilation rate of 50%. The resulting EOM powders were stored at −20 °C in darkness until use.

### 2.3. Preparation of Starch-Based Bioplastics

All formulations were prepared using 100 mL of distilled water. The film-forming matrix consisted of 5.94 g of lucuma seed starch and 2.00 mL of glycerol. Treatments containing microencapsulated essential oil incorporated 598.29 mg of EOM, whereas formulations containing free EO received 80 µL of EO, corresponding to the amount of EO present in EOM.

Initially, the starch suspension was heated at 80 °C under constant stirring (450 rpm) until complete gelatinization was achieved. During heating, beakers were covered with aluminum foil to minimize water loss by evaporation. Subsequently, the temperature was reduced to 60 °C, glycerol was added, and stirring speed was increased to 1000 rpm for 15 min to ensure homogenization. After an additional 10 min, EO or EOM was incorporated according to each experimental treatment. Finally, 1 mL of acetic acid was added to all formulations to improve dispersion and homogenization of the film-forming solutions. The resulting mixtures were cast into molds (10 × 15 cm) and dried at 45 ± 2 °C for 24 h under 70% relative humidity to obtain the starch-based bioplastics.

Functionally, the selected formulations exhibited water vapor permeability (WVP) values ranging from 2.75 to 3.48 g·mm·h^−1^·m^−2^·kPa^−1^ and oxygen permeability (OP) values between 4.07 and 5.68 g·m^−2^·h^−1^, depending on whether free or microencapsulated EO was incorporated. These permeability ranges were considered suitable for evaluating the ability of the films to regulate moisture loss and gas exchange during strawberry storage.

A summary of the principal physicochemical and barrier properties of the bioplastics is presented in Table 1, whereas the complete characterization and analytical methodologies are available in Mena-Chacon et al. [13] and in the Supplementary Materials.

### 2.4. Validation Using Strawberries as a Food Model

Bioplastics were prepared in molds (10 × 15 cm) and heat-sealed to obtain packaging bags. Four packaging treatments were evaluated: (i) control bioplastic film without active compounds, (ii) bioplastic film containing free EO, (iii) bioplastic film containing EOM, and (iv) a commercial low-density polyethylene (LDPE) bag used as a reference.

**Figure 1:**
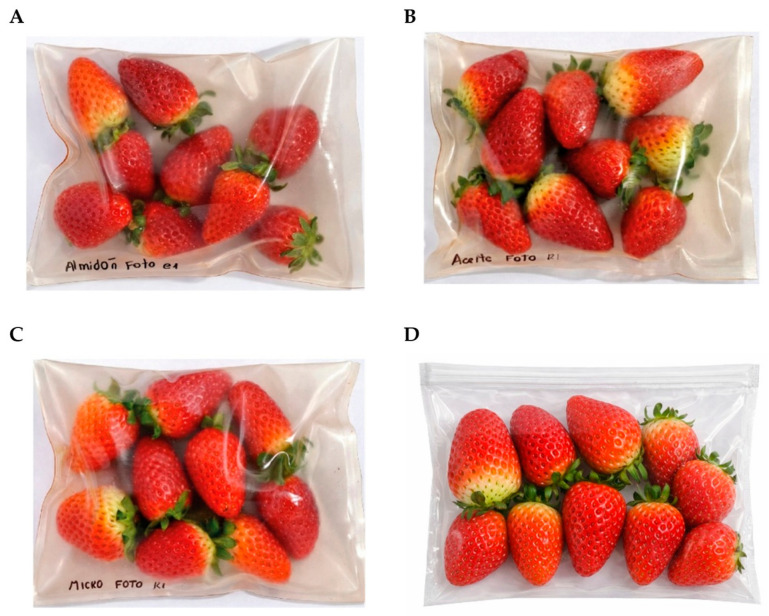
Visual appearance of strawberries packaged in different films: (**A**) lucuma seed starch-based bioplastic (Control; labeled *“Almidón”* in the original photograph), (**B**) bioplastic containing free essential oil (labeled *“Aceite”*), (**C**) bioplastic containing microencapsulated essential oil (labeled *“Micro”*), and (**D**) LDPE.

All bags, including LDPE, were microperforated with five circular holes (0.33 mm diameter), corresponding to a ventilation area of 0.0057%. Strawberries ‘San Andreas’ were packaged at an average weight of 100 g per bag and stored at 4 ± 0.5 °C and ≥80% relative humidity for 16 days. The internal atmosphere (CO_2_ and O_2_ concentrations) was monitored using a gas analyzer (AMETEX Mocon, Minneapolis, MN, USA). From day 2 onward, all treatments exhibited stable internal gas composition, with values close to atmospheric conditions (CO_2_ ≈ 0.2–0.3% and O_2_ ≈ 20.6%), thereby indicating that micro-perforation effectively prevented the development of modified atmospheres [34,35]. Fruit quality attributes and postharvest losses were evaluated as 0, 3, 9, 13, and 16 days of storage. At each sampling time, three bags per treatment were analyzed as independent replicates. Initial quality evaluations were performed immediately after harvest (day 0). Representative visual appearance of strawberries under each packaging treatment is shown in Figure 1.

#### 2.4.1. Weight Loss, Total Soluble Solids, pH, and Titratable Acidity

Weight loss was determined by recording fruit mass at each sampling time and expressed as percentage loss relative to the initial weight. Total soluble solids (TSS) were measured by homogenizing 50 g of strawberry fruit for 30 s, placing a drop of the juice on a digital refractometer (RHB-32ATC, Huake Instrument Co., Ltd., Shenzhen, China), and expressing the results as °Brix. The pH was measured directly in the filtered strawberry juice using a digital pH meter (Model PH1300, LAQUA, Horiba Advanced Techno Co., Ltd., Kyoto, Japan). Titratable acidity (TA) was determined by titration with 0.1 N sodium hydroxide using phenolphthalein as an indicator and expressed as percentage of citric acid [9,25].

#### 2.4.2. Firmness

Fruit firmness was evaluated by a compression test using a texture analyzer (CT3, Brookfield Engineering Labs, Inc., Middleboro, MA, USA) equipped with a 10 kg load cell and a cylindrical probe (2 mm diameter). Tests were performed at a crosshead speed of 2 mm·s^−1^ to a deformation distance of 10 mm [9]. Data acquisition and analysis were conducted using TexturePro software (version 1.0.19).

#### 2.4.3. External Color

Color parameters L* (lightness), a* (green–red), and b* (blue–yellow) were determined using a portable colorimeter (TES 135 A, TES Electrical Electronic Corp., Taipei, Taiwan) and expressed in the CIE Lab* color space [9]. Two measurements were taken on opposite sides of each fruit. Total color difference (ΔE) was calculated according to the following equation.

$$∆E=\sqrt{{{(∆L}^{*})}^{2}+{{(∆a}^{*})}^{2}+{{(∆b}^{*})}^{2}}$$

where ΔL*, Δa*, and Δb* represent the changes in L*, a*, and b* values of stored fruit at a given sampling time relative to fresh fruit at harvest (day 0). Results were expressed as mean ± standard error.

#### 2.4.4. Antioxidant Capacity, Total Phenolics, and Total Anthocyanins

Methanolic extracts were prepared from fresh strawberry samples at a 1:10 (*w*/*v*) ratio using 80% methanol. The mixtures were shaken at 300 rpm for 1 h in an orbital shaker (LAUDA, Lauda-Königshofen, Germany), centrifuged at 25,400× *g* for 15 min at 4 °C, and filtered through Whatman No. 40 paper. Quantification of bioactive compounds was performed according to Quispe-Sanchez et al. [36].

Antioxidant capacity was determined spectrophotometrically at 517 nm and expressed as µmol Trolox equivalents (TE) per 100 g fresh weight (FW). Total phenolic content was measured at 765 nm and expressed as mg gallic acid equivalents (GAE) per 100 g FW. Total anthocyanins were quantified at 535 nm in the crude extract and expressed as mg cyanidin-3-glucoside (C3G) equivalents per 100 g FW. Calibration curves showed linearity within the working range (R^2^ > 0.99).

#### 2.4.5. Decay Incidence

Decay incidence was evaluated as the percentage of fruits showing visible symptoms of fungal decay relative to the total number of fruits per treatment at each evaluation time [37].

### 2.5. Statistical Analysis

Postharvest quality parameters were analyzed using a two-way analysis of variance (two-way ANOVA), considering packaging treatment and storage time as fixed factors, including their interaction effect (treatment × storage time). This approach allowed evaluation of both the individual and combined effects of packaging system and storage duration on strawberry quality attributes during refrigerated storage. When significant differences were detected, mean comparisons were performed using Tukey’s honestly significant difference (HSD) test at a significance level of *p <* 0.05. Prior to analysis, data were evaluated for normality and homogeneity of variances using the Shapiro–Wilk and Levene tests, respectively.

In addition, Pearson’s correlation analysis was performed to evaluate relationships among physicochemical, colorimetric, bioactive, and decay-related variables. Principal component analysis (PCA) was also conducted to identify multivariate patterns associated with packaging treatments and storage behavior of strawberries during refrigerated storage. Statistical analyses and graphical representations were performed using RStudio v. 4.4.1. (R Core Team, Vienna, Austria) and OriginPro 2025 software (OriginLab Corporation, Northampton, MA, USA).

## 3. Results

### 3.1. Postharvest Performance of Strawberries Packed with Starch-Based Bioplastics

The two-way ANOVA revealed that packaging treatment significantly affected all evaluated variables, highlighting the strong influence of the packaging system on the physicochemical, colorimetric, functional, and microbiological behavior of strawberries during refrigerated storage (Table 2). In contrast, the effect of storage time and the treatment × storage time interaction varied depending on the parameter evaluated.

Variables associated with fruit deterioration dynamics, including weight loss, total soluble solids, antioxidant capacity, total phenolics, total anthocyanins, and decay incidence, exhibited significant effects for both main factors and their interaction. This indicates that the response of these parameters depended not only on storage duration but also on the specific packaging system applied throughout storage.

Conversely, several color-related parameters, including L*, a*, b*, hue angle, and ΔE, did not show significant treatment × storage time interactions, suggesting that temporal changes in these variables were not differentially modulated by the packaging systems. However, packaging treatment exerted a highly significant main effect on all color attributes (*p <* 0.0001), highlighting the predominant influence of packaging type on strawberry visual quality during storage. Similarly, several variables were not significantly influenced by storage time alone, while packaging treatment exerted significant effects on these parameters, suggesting that the packaging system was the primary factor associated with the observed differences under the evaluated storage conditions.

### 3.2. Weight Loss

**Figure 2:**
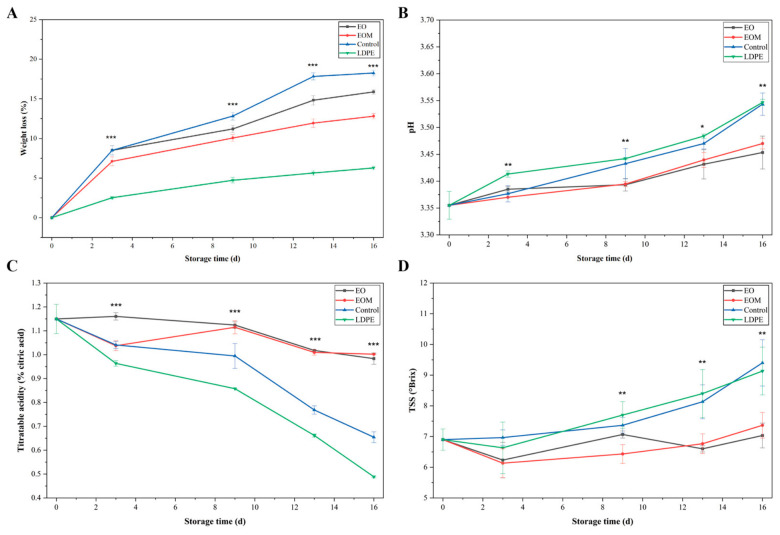
Changes in physicochemical quality parameters of strawberries ‘San Andreas’ during cold storage (4 ± 0.5 °C; ≥80% RH) under different packaging systems: (**A**) weight loss (%), (**B**) pH, (**C**) titratable acidity (% citric acid), and (**D**) total soluble solids (TSS, °Brix). Treatments included conventional LDPE packaging, lucuma seed starch-based bioplastic without essential oil (Control), bioplastic containing free lemon verbena essential oil (EO; lemon verbena), and bioplastic containing microencapsulated essential oil (EOM). Weight loss values were calculated relative to the initial fruit weight at day 0. Data are expressed as mean ± standard deviation (*n* = 3). Asterisks indicate significant differences among treatments at each storage time according to Tukey’s HSD test (* *p <* 0.05; ** *p <* 0.01; *** *p <* 0.001).

Weight loss increased progressively in all treatments throughout refrigerated storage (Figure 2A). Two-way ANOVA revealed significant effects of packaging treatment, storage time, and their interaction on strawberry weight loss (*p <* 0.0001; Table 2).

Strawberries packaged in LDPE exhibited the lowest weight loss during the entire storage period, reaching final values of 6.29 ± 0.22% after 16 d. In contrast, fruits packaged in lucuma starch-based bioplastics showed significantly higher weight loss values. Among the biodegradable formulations, the control treatment exhibited the highest final weight loss (18.25 ± 0.35%), followed by EO (15.88 ± 0.28%), whereas EOM showed comparatively lower values (12.81 ± 0.34%). Differences among treatments became more evident as storage progressed, particularly from day 9 onward, consistent with the significant treatment × storage time interaction detected by ANOVA (Table 2).

### 3.3. Total Soluble Solids, pH, and Titratable Acidity

TSS and pH increased during storage, whereas TA decreased in all treatments (Figure 2B–D). Two-way ANOVA revealed significant effects of packaging treatment and storage time on these parameters (*p <* 0.0001; Table 2). LDPE-packaged strawberries exhibited the greatest increases in TSS and pH during storage, reaching 9.16 °Brix and pH 3.54 after 16 d, respectively. In contrast, strawberries packaged in EO and EOM bioplastics showed comparatively lower increases in both parameters throughout storage. TA decreased progressively in all treatments, although the magnitude of reduction differed among packaging systems. LDPE exhibited the greatest decline in TA, decreasing from 1.15 to 0.49% citric acid after 16 d, whereas EO and EOM treatments maintained comparatively higher acidity values throughout storage.

### 3.4. Firmness

**Figure 3:**
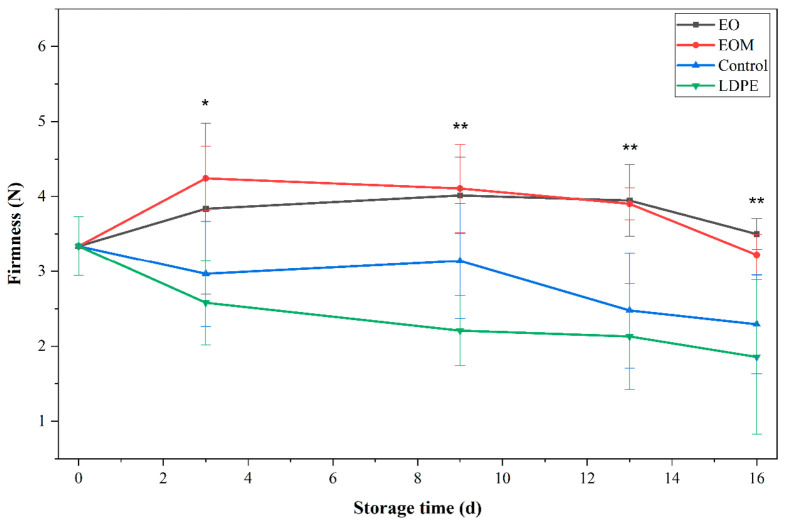
Changes in firmness of strawberries ‘San Andreas’ during cold storage (4 ± 0.5 °C; ≥80% RH) under different packaging systems. Treatments included LDPE, lucuma seed starch-based bioplastic (Control), bioplastic with free lemon verbena essential oil (EO; lemon verbena), and bioplastic with microencapsulated essential oil (EOM). Data are expressed as mean ± SD (*n* = 3). Asterisks indicate significant differences among treatments at each storage time according to Tukey’s test (* *p <* 0.05; ** *p <* 0.01).

Firmness decreased progressively during refrigerated storage in all treatments (Figure 3). Two-way ANOVA revealed significant effects of packaging treatment (*p <* 0.0001) and storage time (*p =* 0.0041) on strawberry firmness, whereas the treatment × storage time interaction was not significant (*p =* 0.9375) (Table 2).

Across storage, strawberries packaged in EO and EOM bioplastics maintained significantly higher firmness values than those packaged in the Control and LDPE treatments according to Tukey’s test (*p <* 0.05). Mean firmness values were 3.82 and 3.87 N for EO and EOM, respectively, compared with 2.72 and 2.19 N for Control and LDPE. Regarding storage duration, fruits stored for 16 d exhibited significantly lower firmness values than those evaluated at earlier storage times. Although firmness decreased in all treatments throughout storage, EOM showed the highest firmness values at most evaluation times (Figure 3).

### 3.5. Color

Color parameters were significantly influenced by packaging treatment, whereas storage time and the treatment × storage time interaction did not significantly affect L*, a*, b*, hue angle, or ΔE values (Table 2). In contrast, chroma (C*) was significantly affected by packaging treatment, storage duration, and their interaction (*p <* 0.05).

**Figure 4:**
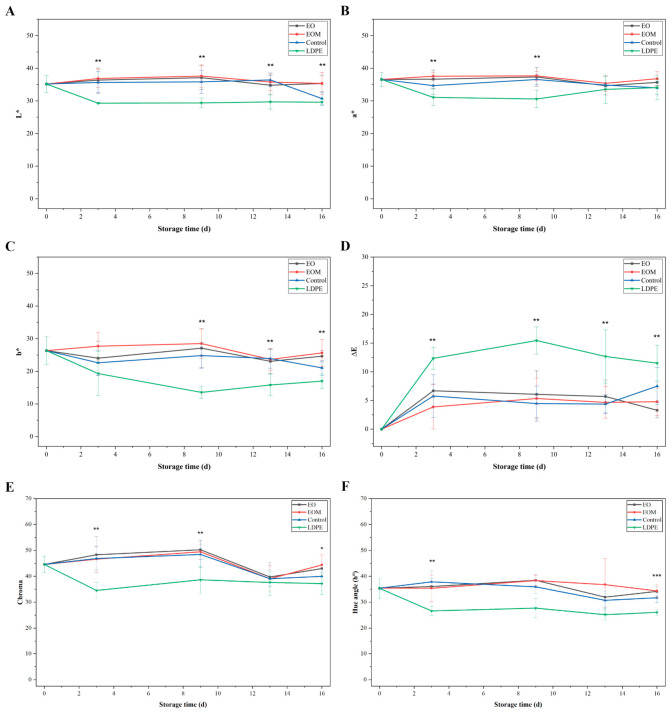
Changes in color parameters of strawberries ‘San Andreas’ during cold storage (4 ± 0.5 °C; ≥80% RH) under different packaging systems: (**A**) L*, (**B**) a, (**C**) b*, (**D**) total color difference (ΔE), (**E**) chroma, and (**F**) hue angle (h°). Treatments included LDPE, lucuma seed starch-based bioplastic (Control), bioplastic with free lemon verbena essential oil (EO; lemon verbena), and bioplastic with microencapsulated essential oil (EOM). Data are expressed as mean ± SD (*n* = 3). Asterisks indicate significant differences among treatments within each storage time according to Tukey’s test (* *p <* 0.05; ** *p <* 0.01; *** *p <* 0.0001).

Among treatments, LDPE-packaged strawberries exhibited significantly lower L*, a*, b*, and hue values compared with strawberries packaged in starch-based bioplastics (*p <* 0.05). Conversely, EOM and EO treatments maintained the highest mean values for these parameters throughout storage (Figure 4A–D). Although slight fluctuations were observed during storage, no significant main effects of storage time were detected for L*, a*, b*, hue angle, or ΔE according to two-way ANOVA (*p >* 0.05; Table 2).

Regarding ΔE, LDPE exhibited significantly higher values than all starch-based bioplastic treatments, reaching a mean value of 12.99, whereas EOM showed the lowest ΔE values (4.67), followed by EO (5.44) and the Control treatment (5.53). Chroma values exhibited significant temporal variations depending on the packaging system (Figure 4E). EO and EOM treatments maintained the highest chroma values during storage, particularly at day 9, whereas LDPE showed comparatively lower values throughout storage (Figure 4).

### 3.6. Bioactive-Related Quality

Antioxidant capacity, total phenolics, and total anthocyanins were significantly affected by packaging treatment, storage time, and their interaction (*p <* 0.0001; Table 2), indicating that temporal changes in bioactive-related parameters differed among packaging systems.

**Figure 5:**
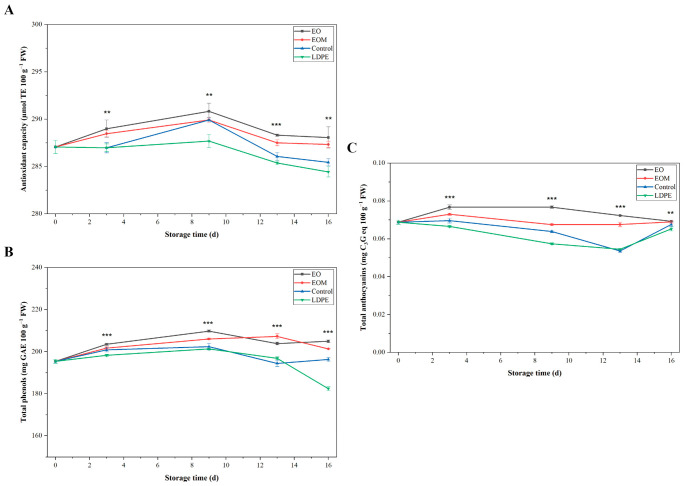
Changes in bioactive-related quality attributes of strawberries ‘San Andreas’ during cold storage (4 ± 0.5 °C; ≥80% RH) under different packaging systems: (**A**) antioxidant capacity, (**B**) total phenolic content, and (**C**) total anthocyanins. Treatments included LDPE, lucuma seed starch-based bioplastic (Control), bioplastic with free lemon verbena essential oil (EO; lemon verbena), and bioplastic with microencapsulated essential oil (EOM). Data are expressed as mean ± SD (*n* = 3). Asterisks indicate significant differences among treatments at each storage time according to Tukey’s test (** *p <* 0.01; *** *p <* 0.0001).

EO and EOM treatments maintained higher antioxidant capacity values throughout storage compared with Control and LDPE treatments (Figure 5A). According to Tukey’s test, EO exhibited the highest overall antioxidant capacity (289.05 µmol TE/100 g FW), followed by EOM (288.30 µmol TE/100 g FW), whereas LDPE showed the lowest mean values (286.12 µmol TE/100 g FW).

Similarly, total phenolic content was significantly higher in EO and EOM treatments than in Control and LDPE (Figure 5B). EO showed the highest mean phenolic content (205.51 mg GAE/100 g FW), while LDPE exhibited the lowest values (194.69 mg GAE/100 g FW). At day 16, LDPE-packaged strawberries showed the lowest phenolic concentration (182.38 mg GAE/100 g FW), whereas EO maintained comparatively higher values (204.94 mg GAE/100 g FW).

Total anthocyanin content also varied significantly depending on packaging treatment and storage duration (Figure 5C). EO and EOM treatments maintained higher anthocyanin levels throughout storage, whereas LDPE and Control treatments exhibited lower concentrations at several evaluation times. The highest anthocyanin values were observed in EO at days 3 and 9 (0.08 mg C_3_G eq·100 g^−1^ FW), while the lowest values were detected in LDPE and Control treatments at day 13 (0.05 mg C_3_G eq·100 g^−1^ FW).

### 3.7. Decay Incidence

**Figure 6:**
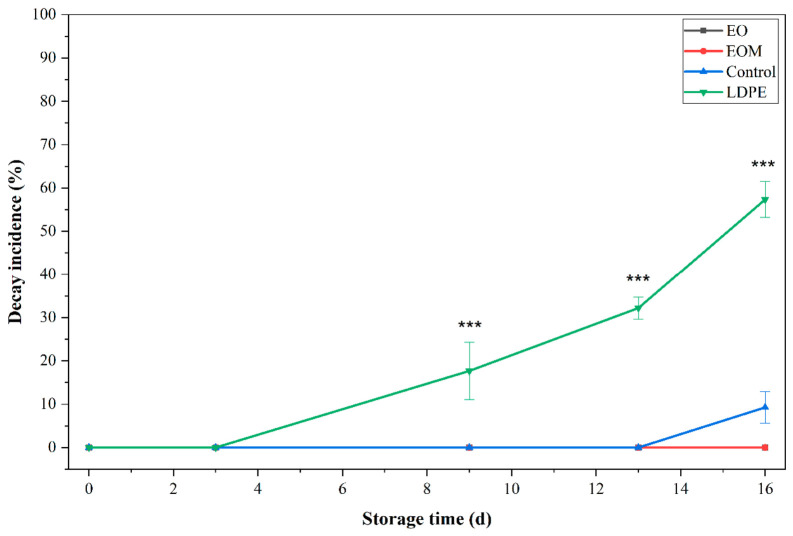
Decay incidence of strawberries ‘San Andreas’ during cold storage (4 ± 0.5 °C; ≥80% RH) under different packaging systems. Treatments included LDPE, lucuma seed starch-based bioplastic (Control), bioplastic with free lemon verbena essential oil (EO; lemon verbena), and bioplastic with microencapsulated essential oil (EOM). Data are expressed as mean ± SD (*n* = 3). Asterisks indicate significant differences among treatments at each storage time according to Tukey’s test (*** *p <* 0.0001).

Decay incidence was significantly affected by packaging treatment, storage time, and their interaction (*p* < 0.0001; Table 2). Disease development increased progressively during storage, although marked differences were observed among packaging systems (Figure 6).

LDPE-packaged strawberries exhibited the highest decay incidence throughout storage, reaching 57.34% after 16 d. In contrast, EO and EOM treatments maintained complete decay suppression during the entire evaluation period, with no visible fungal incidence detected in any replicate.

The Control treatment showed intermediate behavior, with decay incidence remaining at 0% until day 13 and increasing to 9.25% at day 16. According to Tukey’s test, LDPE exhibited significantly higher decay incidence than all starch-based bioplastic treatments (*p* < 0.05), whereas EO and EOM did not differ significantly from each other and maintained the lowest mean values throughout storage.

### 3.8. Multivariate Analysis

**Figure 7:**
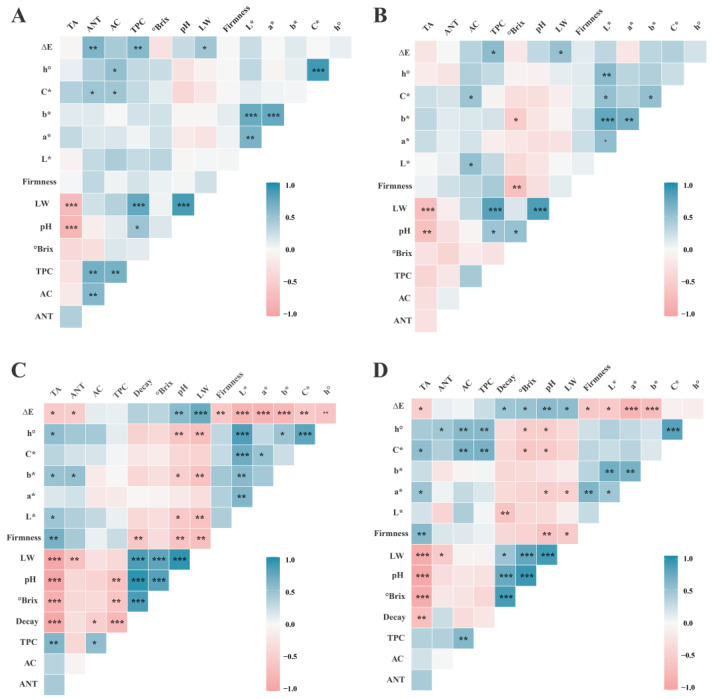
Pearson correlation heatmaps showing the relationships among physicochemical and quality-related parameters of strawberries under different packaging systems: (**A**) control bioplastic (EO), (**B**) bioplastic with free essential oil (EOM), (**C**) bioplastic with microencapsulated essential oil (LDPE), and (**D**) low-density polyethylene (Control). LW: weight loss; ΔE: total color difference; ANT: anthocyanins; TA: titratable acidity; AC: antioxidant capacity; TPC: total phenolic content. Color intensity represents the strength and direction of the correlation (blue: positive; red: negative; scale from −1 to +1). Asterisks indicate statistically significant correlations (* *p <* 0.05; ** *p <* 0.01; *** *p <* 0.001).

Pearson correlation analysis revealed consistent and biologically meaningful relationships among physicochemical and quality attributes of strawberries (Figure 7). Strong negative correlations were observed between titratable acidity and key deterioration indicators, including pH (r = −0.97), decay incidence (r = −0.94), and total soluble solids (°Brix) (r = −0.84), indicating that the loss of acidity is closely associated with senescence progression. Conversely, titratable acidity showed positive correlations with firmness (r = 0.69), total phenolic content (r = 0.58), and antioxidant capacity (r = 0.43), suggesting that higher acidity is linked to better structural integrity and preservation of bioactive compounds (Supplementary Table S1).

Weight loss (LW) was also closely associated with ripening and quality changes. LW showed a strong positive correlation with pH (r = 0.84) and a strong negative correlation with titratable acidity (r = −0.68), reinforcing the link between water loss and metabolic progression during storage. In addition, LW was positively correlated with total phenolic content (r = 0.80) and total color difference (ΔE) (r = 0.50), suggesting that dehydration processes were accompanied by biochemical and visual modifications in the fruit.

Decay incidence was strongly associated with quality deterioration, showing positive correlations with pH (r = 0.93) and °Brix (r = 0.86), and negative correlations with firmness (r = −0.60) and TPC (r = −0.73). These relationships confirm that microbial spoilage is closely coupled with metabolic changes and the depletion of protective compounds during storage.

Color parameters were strongly interrelated, with lightness (L*) showing high positive correlations with hue angle (h°) (r = 0.86) and chroma (C*) (r = 0.72), and strong negative correlations with total color difference (ΔE) (r = −0.83), indicating that visual quality degradation is directly associated with pigment destabilization. Additionally, ΔE exhibited negative correlations with firmness (r = −0.63) and bioactive-related variables such as anthocyanins (r = −0.60), reflecting the link between visual changes and overall fruit deterioration.

**Figure 8:**
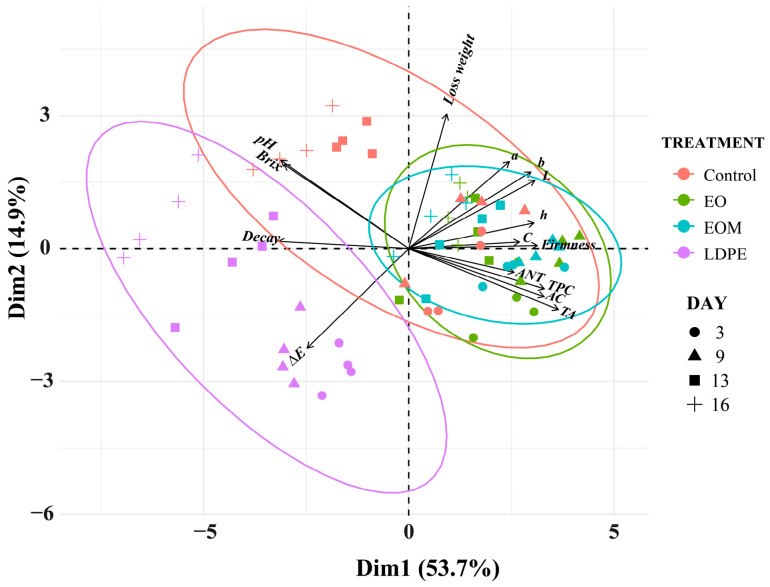
Principal component analysis of the physicochemical properties of strawberries under different packaging treatments during storage. ΔE: total color difference; ANT: anthocyanins; TA: titratable acidity; AC: antioxidant capacity; TPC: total phenolic content.

PCA explained 68.6% of the total variance, with PC1 and PC2 accounting for 53.7% and 14.9% of the variation, respectively (Figure 8). The first two components clearly separated treatments according to their preservation performance. EO and EOM treatments clustered with variables associated with quality maintenance, including firmness, titratable acidity, total phenolic content, antioxidant capacity, anthocyanins, and color-related attributes. In contrast, LDPE was associated with pH, °Brix, decay incidence, and ΔE, indicating a stronger association with senescence and deterioration processes. Weight loss was positioned separately from decay-related variables, suggesting that moisture loss alone was not the primary determinant of overall quality deterioration.

The EOM treatment exhibited a more compact clustering pattern than the other treatments, indicating greater consistency in the preservation of multiple quality attributes throughout storage. Overall, the multivariate analysis highlights that postharvest quality was governed by tightly interconnected metabolic, structural, biochemical, and microbiological processes and demonstrates that active starch-based bioplastics containing lemon verbena essential oil effectively modulated these interactions to delay fruit deterioration during refrigerated storage.

## 4. Discussion

### 4.1. Moisture Regulation and Weight Loss Dynamics

The progressive increase in weight loss observed during refrigerated storage (Figure 2A) reflects the combined effects of fruit transpiration, respiration, and package permeability. Two-way ANOVA revealed significant effects of packaging treatment, storage time, and their interaction (*p* < 0.0001; Table 2), indicating that differences among packaging systems became more pronounced as storage progressed, particularly after day 9.

As expected, strawberries packaged in LDPE exhibited the lowest weight loss throughout storage, reaching final values of 6.29 ± 0.22% after 16 d. This behavior is consistent with the low water vapor permeability typically associated with conventional polyolefin films, which efficiently restrict moisture transfer between the fruit and the external environment [32,38,39]. However, this superior barrier performance did not translate into better overall preservation of strawberry quality, suggesting that excessive moisture retention may negatively affect other postharvest attributes.

In contrast, all starch-based bioplastics exhibited significantly higher weight loss values than LDPE, ranging from 12.81% in the EOM treatment to 18.25% in the control formulation. This behavior is consistent with the hydrophilic nature of starch matrices, which facilitates water vapor diffusion compared with hydrophobic synthetic polymers such as LDPE [6,40]. Notably, the incorporation of lemon verbena essential oil reduced weight loss relative to the control bioplastic, particularly when incorporated in microencapsulated form. Final weight loss values reached 15.88% for EO and 12.81% for EOM, compared with 18.25% in the control treatment. The superior performance of EOM suggests that microencapsulation improved the ability of the polymer matrix to regulate moisture transfer during storage.

Several non-exclusive mechanisms may explain the improved moisture retention observed in the EOM treatment. Microencapsulation may increase the effective tortuosity of the polymer matrix, generating longer diffusion pathways for water vapor molecules and thereby reducing net vapor flux [41,42]. Additionally, the incorporation of microcapsules can modify film microstructure and barrier performance by altering matrix organization and thickness [43,44]. Similar improvements in moisture regulation have been reported in active packaging systems containing encapsulated bioactive compounds, where enhanced water retention is associated with controlled mass transfer rather than with absolute barrier properties alone [29,44].

Although none of the starch-based formulations reached the low weight loss levels observed in LDPE, this behavior may not necessarily compromise fresh produce preservation. Excessively restrictive barriers can create highly saturated internal atmospheres that favor condensation, microbial proliferation, and anaerobic respiration, ultimately accelerating quality deterioration [8,10]. Conversely, excessive permeability, as observed in the control treatment, promotes rapid dehydration and loss of commercial quality. Therefore, the effectiveness of packaging systems for fresh produce appears to depend on achieving a balanced mass transfer, sufficiently low to reduce dehydration while allowing adequate gas exchange compatible with fruit metabolism. Among the evaluated formulations, the EOM treatment appears to provide the most favorable balance between moisture retention and physiological stability during refrigerated storage.

### 4.2. Influence of Packaging on Metabolic Activity

The evolution of TSS, pH, and TA reflects the metabolic progression of strawberries during postharvest storage [10]. In general, TSS and pH increased while TA decreased throughout refrigerated storage (Figure 2B–D), consistent with the typical ripening and senescence behavior reported for strawberries during postharvest storage [45,46].

Two-way ANOVA revealed significant effects of packaging treatment; storage time; and their interaction on TSS, pH, and TA (*p <* 0.0001; Table 2), indicating that the metabolic evolution of the fruit was strongly influenced by the packaging system.

Strawberries packaged in LDPE exhibited the most pronounced physicochemical changes, reaching the highest final values of TSS (9.16 °Brix) and pH (3.54), together with the lowest TA value (0.49% citric acid) after 16 d of storage. Although LDPE effectively reduced moisture loss (Section 4.1), these results suggest accelerated organic acid consumption and advanced metabolic progression during storage. Similar trends have been associated with intensified respiratory activity and senescence-related biochemical transformations in stored strawberries [8,10,47].

In contrast, strawberries packaged in lucuma starch-based bioplastics exhibited comparatively slower changes in TSS, pH, and TA, indicating moderated metabolic activity during storage. Among these treatments, the control bioplastic showed greater increases in TSS and pH than EO and EOM, whereas formulations containing essential oil maintained comparatively higher acidity values throughout storage.

Notably, the EOM treatment consistently showed the slowest physicochemical changes among the biodegradable formulations. After 16 d, EOM-packaged strawberries maintained lower TSS accumulation and TA values closer to the initial levels compared with EO. Although the absolute differences between EO and EOM were moderate, the same trend was consistently observed across all three metabolic indicators, suggesting a stabilizing effect associated with the incorporation of microencapsulated essential oil.

Several mechanisms may explain the improved performance of the EOM treatment. The controlled release of essential oil compounds from the microcapsules likely prolonged the availability of antioxidant and antimicrobial components during storage, whereas free EO may have been more susceptible to volatilization and rapid depletion [23]. In addition, microencapsulation may have contributed to a more stable film microstructure, influencing gas transfer dynamics and moderating respiratory metabolism [42,43]. Similar responses have been reported in active packaging systems containing encapsulated bioactive compounds, where delayed metabolic progression has been associated with combined barrier-related and antioxidant effects [44,48].

Because film microperforation prevented the establishment of a modified atmosphere, the observed effects are mainly attributable to passive regulation of gas exchange through film permeability rather than to active atmospheric modification [32,49,50]. Overall, these results suggest that the incorporation of microencapsulated essential oil contributed to maintaining a more stable physicochemical state during strawberry storage.

### 4.3. Structural Integrity and Firmness Preservation

Firmness decreased progressively in all treatments during refrigerated storage (Figure 3). Two-way ANOVA revealed significant effects of packaging treatment (*p <* 0.0001) and storage time (*p =* 0.0041) on strawberry firmness, whereas the treatment × storage time interaction was not significant (*p =* 0.9375; Table 2). This indicates that the relative performance of the packaging systems remained consistent throughout storage.

A notable relationship emerged when comparing firmness behavior with weight loss dynamics (Section 4.1). Despite exhibiting the lowest weight loss (6.29%), strawberries packaged in LDPE showed the lowest mean firmness values (2.19 N). In contrast, the control bioplastic treatment maintained significantly higher firmness (2.72 N) despite exhibiting substantially greater water loss (18.25%). These results indicate that moisture retention alone is insufficient to preserve tissue structure and suggest that the microenvironment generated by the packaging system plays a critical role in fruit softening.

The pronounced firmness loss observed in LDPE-packaged strawberries may be associated with accelerated senescence-related processes under highly restrictive barrier conditions. Excessive limitation of gas exchange can promote metabolic imbalances and favor the enzymatic degradation of cell wall components, including pectins and structural polysaccharides, even when dehydration is reduced [51,52]. Similar responses have been reported in strawberries stored under high-humidity or low gas-exchange conditions, where tissue softening progresses despite limited transpiration.

In contrast, strawberries packaged in starch-based bioplastics containing lemon verbena essential oil (EO and EOM) maintained significantly higher firmness values than both LDPE and the control bioplastic (*p <* 0.05). Mean firmness values reached 3.82 and 3.87 N for EO and EOM, respectively, compared with 2.72 N for the control treatment and 2.19 N for LDPE. No significant differences were observed between EO and EOM, indicating that the incorporation of essential oil itself was the main factor associated with improved firmness retention.

Several mechanisms may explain the enhanced structural preservation observed in EO- and EOM-containing bioplastics. Essential oil compounds may contribute to delaying softening by reducing oxidative stress and limiting senescence-associated degradation of cell wall polymers [44,48,53]. In addition, as discussed in Section 4.1 and Section 4.2, the moderate permeability of starch-based bioplastics likely promoted a more balanced regulation of water vapor and gas exchange, generating storage conditions compatible with slower metabolic progression.

Although microencapsulation did not provide a statistically significant additional effect on firmness preservation relative to free EO, the EOM treatment exhibited superior performance in other quality attributes. Specifically, EOM reduced weight loss more effectively and better preserved titratable acidity and soluble solids during storage. These results suggest that the microencapsulated formulation provided broader stabilization of strawberry quality, likely associated with the sustained release of bioactive compounds throughout refrigerated storage [29,44].

### 4.4. Color Stability and Pigment Preservation

Color parameters are critical indicators of visual quality and consumer acceptability in fresh strawberries. Two-way ANOVA revealed that packaging treatment significantly affected all evaluated color attributes, whereas storage time and the treatment × storage time interaction were not significant for L*, a*, b*, hue angle, or ΔE (*p >* 0.05; Table 2). These results indicate that refrigerated storage at 4 °C effectively limited progressive color deterioration over time, while the packaging system itself exerted the main influence on visual quality preservation. In contrast, chroma (C*) exhibited significant effects of treatment, storage time, and their interaction (*p <* 0.05), indicating that color saturation was more responsive to storage conditions than the other color parameters.

Among the evaluated systems, LDPE-packaged strawberries exhibited significantly lower L*, a*, b*, and hue values than all starch-based bioplastic treatments, indicating darker fruits with lower visual quality. This behavior is consistent with the pronounced firmness loss observed in LDPE-treated strawberries (Section 4.3) and suggests that the restrictive microenvironment generated by this material may favor oxidative processes affecting pigment stability, even under refrigerated conditions [9].

Conversely, strawberries packaged in EO- and EOM-containing bioplastics maintained higher color parameter values and lower overall visual deterioration throughout storage. Among all evaluated variables, ΔE was the most discriminating indicator of visual preservation. LDPE exhibited the highest ΔE values (12.99), whereas EOM showed the lowest values (4.67), followed by EO (5.44) and the control treatment (5.53). These results indicate that active starch-based bioplastics, particularly EOM, more effectively preserved the original visual appearance of strawberries during refrigerated storage.

The lower ΔE values observed in EO and EOM treatments may be associated with the combined effects of moderated gas exchange and the antioxidant activity of essential oil compounds. As discussed in previous sections, the moderate permeability of starch-based bioplastics likely contributed to maintaining a less oxidative microenvironment than LDPE without excessively restricting gas exchange [53]. In addition, compounds present in lemon verbena essential oil may have contributed to limiting oxidative degradation of anthocyanins and other pigments [22,42]. The slightly lower ΔE values and improved chroma retention observed in EOM suggest that microencapsulation may have enhanced this protective effect through a more sustained release of bioactive compounds during storage [31,54].

Unlike the other color parameters, chroma exhibited significant temporal variation depending on the packaging system. EO and EOM treatments maintained the highest chroma values during storage, particularly at day 9, whereas LDPE showed comparatively lower values throughout storage. This suggests that color saturation was more dynamically affected by storage conditions than the other color attributes.

The strong relationships observed among color parameters, firmness, anthocyanin content, and decay incidence support the close association between pigment stability and overall postharvest quality. Similar relationships between oxidative status, tissue integrity, and pigment preservation have been reported previously in strawberries and other highly perishable fruits [46,55].

### 4.5. Preservation of Bioactive Compounds

The retention of antioxidant capacity, phenolic compounds, and anthocyanins during postharvest storage is strongly influenced by oxidative processes and enzymatic activity. In strawberries, phenolic losses are commonly associated with oxidative reactions catalyzed by polyphenol oxidase (PPO), whereas anthocyanin stability depends on the interaction among oxygen exposure, enzymatic activity, pH, and tissue integrity [56,57]. In the present study, two-way ANOVA revealed significant effects of packaging treatment, storage time, and their interaction on all evaluated bioactive-related parameters (*p <* 0.0001; Table 2), indicating that the temporal evolution of these compounds differed among packaging systems.

Overall, strawberries packaged in EO- and EOM-containing bioplastics maintained higher antioxidant capacity, total phenolic content, and anthocyanin levels throughout storage compared with LDPE and the control bioplastic treatment (Figure 5A–C). Although the overall differences between EO and EOM were moderate, both treatments consistently preserved higher levels of bioactive compounds during storage, particularly at advanced storage stages. For instance, at day 16, LDPE-packaged strawberries exhibited the lowest phenolic content, whereas EO maintained substantially higher values. Similarly, anthocyanin levels were better preserved in EO and EOM treatments throughout storage, supporting the significant treatment × storage time interaction detected by ANOVA.

The greater preservation of bioactive compounds in EO and EOM treatments is consistent with previous reports describing the antioxidant effects of lemon verbena essential oil in strawberries and other fruits [23,25]. Essential oil compounds may contribute to reducing oxidative stress through direct free-radical scavenging activity, thereby limiting the oxidation of phenolic compounds and anthocyanins [22]. In addition, essential oils may suppress oxidative enzymes such as PPO and stimulate antioxidant defense systems involving catalase, peroxidase, and superoxide dismutase, contributing to the maintenance of fruit antioxidant status during storage [25,56,58].

Interestingly, unlike the trends observed for weight loss, titratable acidity, and color stability, microencapsulation did not provide a marked additional advantage over free essential oil for preserving antioxidant capacity, phenolics, or anthocyanins. This behavior may be associated with the distinct kinetics governing these quality attributes. The preservation of bioactive compounds likely depends on the immediate availability of antioxidant molecules capable of scavenging reactive oxygen species during early stages of oxidative stress, whereas gradual release may be more relevant for long-term regulation of physical and metabolic processes such as moisture transfer and respiratory activity. This interpretation is consistent with the temporal patterns observed in the present study, where EO and EOM showed similar performance for bioactive compounds, while the advantages of EOM became more evident for parameters associated with prolonged storage stability.

The preservation of phenolic compounds and anthocyanins in EO and EOM treatments was closely associated with the improved color stability observed during storage (Section 4.4), supporting the strong relationship between pigment preservation and oxidative status in strawberries. Similar responses have been reported previously in starch-based active packaging systems, where the incorporation of natural bioactive compounds contributed to maintaining antioxidant activity, delaying pigment degradation, and extending postharvest quality [59,60]. Overall, these results demonstrate the potential of lucuma starch bioplastics enriched with lemon verbena essential oil as active packaging systems capable of preserving the nutritional and functional quality of fresh strawberries during refrigerated storage.

### 4.6. Control of Microbial Decay

Decay incidence represents one of the most direct indicators of shelf life and commercial acceptability in strawberries. In the present study, two-way ANOVA revealed significant effects of packaging treatment, storage time, and their interaction on decay incidence (*p <* 0.0001; Table 2), indicating that disease development progressed differently among packaging systems during refrigerated storage.

The most remarkable result was the complete suppression of visible fungal decay in strawberries packaged in EO- and EOM-containing bioplastics throughout the entire 16-day storage period. In contrast, LDPE-packaged strawberries exhibited the highest decay incidence, reaching 57.34% at day 16, whereas the control bioplastic treatment showed intermediate behavior, with decay incidence increasing to 9.25% at the end of storage. These results demonstrate that the incorporation of lemon verbena essential oil was the main factor responsible for controlling postharvest fungal development.

According to Tukey’s test, LDPE exhibited significantly higher decay incidence than all starch-based bioplastic treatments (*p <* 0.05), whereas EO and EOM did not differ significantly from each other and maintained complete decay suppression throughout storage. This indicates that, under the evaluated conditions, the presence of essential oil itself—rather than the encapsulation strategy—was sufficient to achieve effective antifungal protection.

The strong antifungal performance observed in EO and EOM treatments is consistent with previous reports describing the inhibitory activity of lemon verbena essential oil against Botrytis cinerea and other postharvest pathogens [24,25,26]. Essential oil compounds may exert antifungal activity through multiple mechanisms, including disruption of fungal cell membranes, interference with respiratory metabolism, and inhibition of ergosterol biosynthesis [22,23]. In addition, the volatile nature of these compounds may facilitate antimicrobial activity within the package headspace, allowing indirect protection of the fruit surface during storage [31,61].

Regarding loss, titratable acidity, and color stability, microencapsulation did not provide additional antifungal protection beyond that achieved with free essential oil. This behavior may be associated with the concentration-dependent nature of antimicrobial activity. The free essential oil likely released sufficient volatile compounds during the early stages of storage to achieve and maintain complete fungal suppression throughout the experimental period, limiting the additional advantage of sustained release from microcapsules. In contrast, parameters associated with long-term metabolic and physical stability may benefit more strongly from gradual release kinetics.

The relationships observed between decay incidence and other quality parameters further support this interpretation. Decay incidence was positively associated with pH and soluble solids content, and negatively associated with firmness and total phenolic content (Figure 7; Supplementary Table S1), indicating that microbial deterioration was closely linked to metabolic progression and the depletion of structural and biochemical defenses during storage.

Similar antimicrobial responses have been reported previously in active packaging systems incorporating essential oils for strawberry preservation under refrigerated conditions [31,42,62]. However, the complete suppression of visible decay achieved for 16 d in the present study highlights the strong potential of lucuma starch bioplastics enriched with lemon verbena essential oil as effective active packaging systems for postharvest strawberry preservation.

### 4.7. Integrated Behavior of Quality Attributes

Pearson correlation analysis and PCA provided an integrated understanding of how packaging systems influenced the interconnected network of quality attributes during refrigerated strawberry storage (Figure 7 and Figure 8; Supplementary Table S1). Strong correlations among titratable acidity, pH, firmness, phenolic compounds, and decay incidence confirmed the close relationship between metabolic progression, structural integrity, oxidative stability, and microbial deterioration during storage.

Color-related variables were also strongly interconnected, particularly L*, hue angle, chroma, and ΔE, supporting the association between visual deterioration, pigment instability, and overall fruit quality loss. In addition, decay incidence was positively associated with pH and soluble solids content, and negatively associated with firmness and phenolic compounds, indicating that microbial spoilage was closely linked to senescence-related metabolic changes and depletion of protective biochemical components. Weight loss was strongly correlated with pH and inversely related to titratable acidity, further indicating that water loss occurred concomitantly with ripening-associated metabolic changes [8,10]. However, its distinct positioning in the PCA space suggests that moisture loss alone did not fully explain the deterioration process, which was more closely associated with the combined effects of metabolic, biochemical, and microbial alterations.

PCA explained 68.6% of the total variance and clearly separated treatments according to their preservation performance (Figure 8). EO and EOM treatments clustered with variables associated with quality maintenance, including firmness, titratable acidity, antioxidant capacity, total phenolics, and anthocyanins, whereas LDPE was associated with variables related to deterioration, such as pH, °Brix, and decay incidence. Interestingly, weight loss was positioned separately from decay- and senescence-related variables in the PCA space, suggesting that moisture loss alone was not the primary determinant of overall postharvest deterioration. This observation further supports the concept that effective strawberry preservation depends on achieving a balanced regulation of mass transfer and metabolic activity rather than maximizing water retention alone.

Notably, the EOM treatment exhibited slightly more compact clustering than EO, suggesting greater consistency in the preservation of multiple quality attributes throughout storage. This behavior may reflect the sustained release of bioactive compounds from microcapsules, which likely promoted more stable regulation of oxidative, metabolic, and microbial deterioration pathways [42,48].

The multivariate analysis demonstrates that the effectiveness of active packaging systems should be evaluated from an integrative perspective, considering their ability to simultaneously modulate interconnected physiological and biochemical processes. Similar coordinated responses involving firmness preservation, bioactive retention, and microbial control have been reported previously in active packaging systems incorporating essential oils for strawberry preservation [31,54]. The present results further demonstrate that lucuma starch bioplastics enriched with lemon verbena essential oil, particularly in microencapsulated form, effectively delayed the overall deterioration trajectory of strawberries during refrigerated storage.

### 4.8. Implications for Active Packaging Design

The findings of this study highlight that effective postharvest preservation requires not only adequate barrier properties, but a balanced integration of permeability and functionality. While conventional packaging systems such as LDPE effectively reduce moisture loss, their limited capacity to regulate the fruit microenvironment and control microbial development restricts their overall performance. This supports the concept that optimal packaging design should aim for controlled mass transfer rather than maximum barrier efficiency [32,49,50].

In this context, lucuma seed starch-based bioplastics incorporating microencapsulated essential oil represent a promising strategy, as they combine sustainable material sources with functional performance. The results indicate that moderate permeability, coupled with the sustained release of bioactive compounds, enables the maintenance of a more stable microenvironment, delaying both physiological deterioration and microbial decay. Similar design principles have been reported in active packaging systems, where the integration of permeability control and bioactive functionality enhances overall preservation outcomes [42,48].

The use of microencapsulation emerges as a key factor in enhancing the stability and effectiveness of active compounds. This is particularly relevant for highly perishable fruits, where continuous antimicrobial and antioxidant protection is required to maintain quality over time [44,53]. Overall, these results support the development of active packaging systems—exemplified by lucuma seed starch bioplastics with microencapsulated lemon verbena essential oil—that integrate controlled permeability and sustained release mechanisms to improve the shelf life and quality of fresh produce under realistic storage conditions.

## 5. Conclusions

This study demonstrates that lucuma (*Pouteria lucuma)* seed starch-based bioplastics incorporating microencapsulated lemon verbena essential oil effectively enhance the postharvest preservation of strawberries during refrigerated storage. Compared with conventional LDPE and non-active bioplastics, the microencapsulated system provided a more balanced regulation of moisture and gas transfer, resulting in improved maintenance of structural, physicochemical, and bioactive attributes, as well as complete suppression of visible decay incidence throughout the 16-day storage period.

The superior performance of the EOM system highlights the importance of integrating controlled permeability with sustained release of bioactive compounds. This combination promotes a more stable fruit microenvironment, delaying deterioration processes without disrupting the natural ripening behavior. These findings support the development of active packaging systems based on biodegradable matrices and controlled-release technologies. In particular, lucuma starch-based bioplastics incorporating microencapsulated essential oil represent a promising strategy for sustainable packaging applications aimed at extending shelf life and preserving the quality of highly perishable fruits under realistic storage conditions. Further studies are needed to evaluate the scalability of this system and its performance under varying temperature regimes.

## Figures and Tables

**Table 1:** Physical properties of bioplastic with lucuma seed starch (control), free lemon verbena essential oil (EO; lemon verbena), and microencapsulated (EOM).

	Control	EO	EOM
Moisture (%)	24.76 ± 0.48	21.01 ± 0.24	18.87 ± 0.45
Water swelling (%)	69.37 ± 2.62	76.94 ± 1.75	71.75 ± 7.37
Solubility (%)	13.30 ± 1.61	10.54 ± 1.37	14.06 ± 1.34
WVP (g mm h^−1^ m^−2^ kPa^−1^)	2.75 ± 0.01	3.45 ± 0.20	3.48 ± 0.14
OP (g m^−2^ h^−1^)	4.07 ± 0.17	5.68 ± 0.17	5.01 ± 0.39
Thickness (mm)	0.205 ± 0.032	0.190 ± 0.013	0.239 ± 0.047

**Table 2:** *p-*values associated with the effects of packaging treatment, storage time, and their interaction on physicochemical, colorimetric, functional, and decay parameters of strawberry fruits cv. ‘San Andreas’.

Source of Variability	Variables														
	Weight Loss	TSS	pH	TA	Firmness	L*	a*	b*	Chroma	hue	∆E	Antioxidant Capacity	Total Phenolics	Total Anthocyanins	Decay Incidence
Treatments	<0.0001	<0.0001	<0.0001	<0.0001	<0.0001	<0.0001	<0.0001	<0.0001	<0.0001	<0.0001	<0.0001	<0.0001	<0.0001	<0.0001	<0.0001
Storage day	<0.0001	<0.0001	<0.0001	<0.0001	0.0041	0.0785	0.6920	0.3501	<0.0001	0.2016	0.6763	<0.0001	<0.0001	<0.0001	<0.0001
Interaction	<0.0001	0.0002	<0.0001	<0.0001	0.9375	0.3794	0.1316	0.2616	0.0282	0.6340	0.3392	<0.0001	<0.0001	<0.0001	<0.0001

## Data Availability

The data presented in this study are available from the corresponding author upon reasonable request. The datasets generated during the current study will be deposited in a public repository upon acceptance of the manuscript.

## Supplementary Materials

The following supporting information can be downloaded at: https://www.mdpi.com/article/10.3390/foods15122093/s1, Supplementary File S1: Methodology for analyzing the physical properties of bioplastics; Supplementary File S2: Pearson correlation coefficients and significance levels used for the correlation matrix and PCA analyses. References [63,64] are cited in the supplementary materials.
