# Supplementary material for: Lucuma Starch-Based Active Packaging Maintains Postharvest Quality of Strawberries During Cold Storage
Source: Foods. 2026 Jun 10;15(12):2093. doi: 10.3390/foods15122093 (PMC13298423; doi:10.3390/foods15122093)
Supplement: Supplementary file 1 [file foods-15-02093-s001.zip › Supplementary File 1.pdf]

Article

Lucuma Starch-Based Active Packaging Maintains Postharvest Quality of Strawberries During Cold Storage

Laydy M. Mena-Chacon [1], Robin Oblitas-Delgado [2], Angel F. Huaman-Pilco [2], Pablo Rituay [1], Krizia Pretell [1],
Eyner Huaman-Huaman [2] and Jonathan Campos [1,3,*]

[1] Escuela de Posgrado, Programa Doctoral en Ciencias para el Desarrollo Sustentable, Facultad de Ingeniería Zootecnista, Agronegocios, Biotecnología y Ciencias de Datos, Universidad Nacional Toribio Rodríguez de Mendoza de Amazonas, Chachapoyas 01001, Peru; laydy.mena@untrm.edu.pe (L.M.M.-C.); pablo.rituay@untrm.edu.pe (P.R.); krizia.pretell@untrm.edu.pe (K.P.)

[2] Grupo de Investigación en Biopesticidas y Bioalternativas para la Protección Vegetal (BIOPEST), Instituto de Investigación para el Desarrollo Sustentable de Ceja de Selva, Universidad Nacional Toribio Rodríguez de Mendoza de Amazonas, Chachapoyas 01001, Peru; robin.oblitas.epg@untrm.edu.pe (R.O.-D.); angel.huaman@untrm.edu.pe (A.F.H.-P.); eyner.huaman@untrm.edu.pe (E.H.-H.)

3 Centro de Investigación Economía Circular y Prospectiva de Agronegocios, Instituto de Investigación en Negocios Agropecuarios, Facultad de Ingeniería Zootecnista, Biotecnología, Agronegocios y Ciencia de Datos, Universidad Nacional Toribio Rodríguez de Mendoza de Amazonas, Chachapoyas, 01001, Peru

* Correspondence: jonathan.campos@untrm.edu.pe

Methodology for analyzing the physical properties of bioplastics

Thickness

Thickness was measured at ten random points using a digital micrometer (0–25 mm range, 0.001 mm resolution; Mitutoyo 293-230), and the average value was used to calculate the cross-sectional area.

Moisture, water swelling, and solubility

Moisture content was determined by oven-drying the samples at 105 °C under natural air circulation until constant mass and expressed as a percentage. Water swelling capacity and solubility were evaluated following the method described by Mouhoub et al. [1], with slight modifications. Briefly, 2 × 2 cm bioplastic samples were immersed in 15 mL of deionized water at room temperature for 24 h under agitation at 120 rpm. After immersion, the samples were gently blotted with filter paper and weighed. Subsequently, the bioplastics were re-dried at 105 °C until constant weight. Swelling ratio (Eq. 1) and water solubility (Eq. 2) were expressed as percentages and calculated from mass changes before immersion, after water uptake, and after re-drying. All analyses were performed in triplicate.

$$Swelling\ ratio\ (\%) = \frac{M_2 - M_1}{M_1} \times 100 \quad \text{Eq 1}$$

$$Water\ solubility\ (\%) = \frac{M_1 - M_3}{M_1} \times 100 \quad \text{Eq 2}$$

where M_1 represents the initial dry mass (g), M_2 the mass after water immersion (g), and M_3 the final dry mass after re-drying (g).

Water vapor permeability (WVP) and oxygen permeability (OP)

WVP was determined according to the ASTM E96-00 standard at 25 °C and 95% relative humidity. Bioplastic-sealed Erlenmeyer flasks were periodically weighed at 24 h intervals over 5 days. WVP was calculated using Eq. 3.

$$WVP = \frac{\Delta m}{A * h * \Delta P} \quad \text{Eq 3}$$

where WVP is the water vapor permeability ($g \cdot mm \cdot h^{-1} \cdot m^{-2} \cdot kPa^{-1}$), Δm is the mass change of the system over time (g), A is the exposed bioplastic area (m^2), h is the time (h), and ΔP is the water vapor pressure difference across the bioplastic (kPa). Thickness (mm) was used for normalization.

OP was evaluated using the oxygen-scavenger absorption method, as described in Chang et al. [2]. Samples were hermetically sealed into test cups containing iron powder as an oxygen absorber and incubated at 25 °C for 48 h. OP was calculated according to Eq. 4.

$$OP = \frac{\Delta m}{A * t} \quad \text{Eq 4}$$

where OP is the oxygen permeability ($g \cdot m^{-2} \cdot h^{-1}$), Δm is the mass change of the test cup before and after exposure (g), t is the exposure time (h), and A is the effective area (m^2).

References:

1. Mouhoub, A.; Guendouz, A.; El Alaoui-Talibi, Z.; Ibnsouda Koraichi, S.; El Modafar, C. Development of Bioactive Formulation Based on Crustacean Chitosan and Thymus Satureioides, Cinnamomum Zeylanicum, and Eugenia Caryophyllus Essential Oils for Food Preservation. *Food Biophys.* **2025**, *20*, doi:10.1007/s11483-025-09981-8.
2. Chang, H.; Zhao, Y.; Zhang, J.; Chen, J.; Yang, T. Slow-Release Antibacterial Film Loaded with Clove Essential Oil Based on Tapioca Starch Used for Bread Preservation. *Food Chem. X* **2025**, *29*, doi:10.1016/j.fochx.2025.102677.
